# Using Machine Learning to Predict Genes Underlying Differentiation of Multipartite and Unipartite Traits in Bacteria

**DOI:** 10.3390/microorganisms11112756

**Published:** 2023-11-13

**Authors:** Fatemah Almalki, Janak Sunuwar, Rajeev K. Azad

**Affiliations:** 1 Department of Biological Sciences and BioDiscovery Institute, University of North Texas, Denton, TX 76203, USA; fatemahalmalki@my.unt.edu (F.A.); janak.sunuwar@ttuhsc.edu (J.S.); 2 Department of Biology, Shaqra University, Al Quwaiiyah 19257, Saudi Arabia; 3 Institute of TeleHealth and Digital Innovation, Texas Tech University Health Sciences Center, Lubbock, TX 79430, USA; 4 Department of Mathematics, University of North Texas, Denton, TX 76203, USA

**Keywords:** bacteria, multipartite genomes, machine learning

## Abstract

Since the discovery of the second chromosome in the *Rhodobacter sphaeroides* 2.4.1 by Suwanto and Kaplan in 1989 and the revelation of gene sequences, multipartite genomes have been reported in over three hundred bacterial species under nine different phyla. This phenomenon shattered the dogma of a unipartite genome (a single circular chromosome) in bacteria. Recently, Artificial Intelligence (AI), machine learning (ML), and Deep Learning (DL) have emerged as powerful tools in the investigation of big data in a plethora of disciplines to decipher complex patterns in these data, including the large-scale analysis and interpretation of genomic data. An important inquiry in bacteriology pertains to the genetic factors that underlie the structural evolution of multipartite and unipartite bacterial species. Towards this goal, here we have attempted to leverage machine learning as a means to identify the genetic factors that underlie the differentiation of, in general, bacteria with multipartite genomes and bacteria with unipartite genomes. In this study, deploying ML algorithms yielded two gene lists of interest: one that contains 46 discriminatory genes obtained following an assessment on all gene sets, and another that contains 35 discriminatory genes obtained based on an investigation of genes that are differentially present (or absent) in the genomes of the multipartite bacteria and their respective close relatives. Our study revealed a small pool of genes that discriminate bacteria with multipartite genomes and their close relatives with single-chromosome genomes. Machine learning thus aided in uncovering the genetic factors that underlie the differentiation of bacterial multipartite and unipartite traits.

## 1. Introduction

The genomes of bacteria were earlier thought to be single circular chromosomes (Jacob et al., 1963 [1]) (Cairns, 1963 [2]) (Bode and Morowitz, 1967 [3]) (Wake, 1973 [4]); however, this view began to change in the past few decades after the identification of a linear chromosome in *Borrelia burgdorferi* in 1989 (Baril et al., 1989 [5]), followed by the report of a secondary chromosome in *Rhodobacter sphaeroides* 2.4.1 and subsequently in dozens of other bacteria from different lineages. The secondary chromosome discovery by Suwanto and Kaplan (Suwanto and Kaplan, 1989, 1992 [6,7]) and by many others in different bacteria, facilitated by the revolution in DNA sequencing technology (Koonin and Wolf, 2008 [8]), firmly established the concept of a multipartite genome structure in bacteriology.

Approximately 10% of all bacteria belonging to different phyla (Val et al., 2014 [9]), including Actinobacteria, Chloroflexi, Deinococcus-Thermus, Firmicutes, and Proteobacteria (α, β, γ), harbor more than one chromosome (Jha et al., 2012 [10]). The multipartite genome architecture was found in approximately 10% of 4541 completed genomes archived in NCBI by 2017 (diCenzo and Finan, 2017 [11]). The conserved “core” genes were also found in secondary or accessory chromosomes, akin to the primary chromosomes. The secondary or accessory chromosomes have a replication and maintenance system similar to that of plasmids or megaplasmids (diCenzo and Finan, 2017 [11]) (Harrison et al., 2010 [12]) (Jiao et al., 2018 [13]) (Misra et al., 2018 [14]). The primary chromosomes have been observed to usually be larger in size than the secondary chromosomes (Prozorov, 2008 [15]). The primary chromosomes harbor essential cellular genes, whereas secondary or accessory chromosomes are often enriched in metabolic genes that help bacteria survive or adapt to specialized niches (Bavishi et al., 2010 [16,17]) (Choudhary et al., 2007 [18]) (Cooper et al., 2010 [19]) (Holden et al., 2004 [20]) (Lykidis et al., 2010 [21]). Secondary chromosomes have also been reported to be disproportionately abundant in “hypothetical” protein-encoding genes (Egan et al., 2005 [22]).

There has been an exponential growth in bacterial genomic data in recent decades for both unipartite and multipartite bacteria. Since the discovery of a secondary chromosome by Suwanto and Kaplan in 1989 (Suwanto and Kaplan, 1989, 1992 [6,7]), hundreds of multipartite genomes have been sequenced and annotated and are now archived among the thousands of bacteria represented in different databases. It is therefore important to develop and apply tools to decipher the genes underlying the versatile traits of these bacteria, including the multipartite and unipartite traits. An important aspect is to understand the genome architectures driving the versatile phenotypes. Machine learning has previously been used to predict the genotypes underlying phenotypes (e.g., antibiotic resistance versus antibiotic susceptibility) (Sunuwar and Azad, 2021, 2022 [23,24]). We therefore assessed machine learning methods to predict the multipartite and unipartite traits, which also made possible the identification of the genes that are potentially responsible for these traits. Here we used supervised machine learning methods to analyze the unipartite and multipartite genomes. This approach was used to determine the genes that discriminate bacteria with multipartite genomes from bacteria with single-chromosome genomes. Our results highlight the significance of machine learning in deciphering evolutionarily and functionally important genes that underlie multipartite traits in bacteria.

## 2. Materials and Methods

### 2.1. Data Collection and Data Preparation

**Figure 1:**
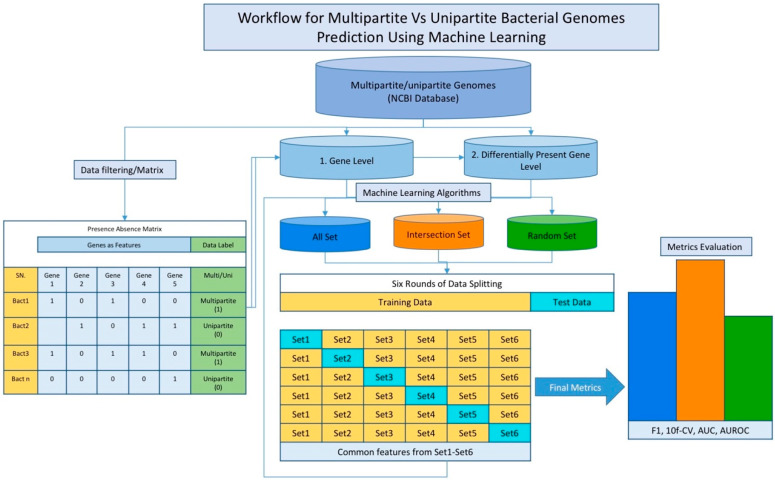
Schematic workflow diagram for applying machine learning to predict genes underlying differentiation of multipartite and unipartite traits in bacteria. The dataset comprising complete multipartite and unipartite genomes downloaded from NCBI was processed for two ML experiments, namely (1) all-gene-level and (2) differentially present gene-level experiments. In the first, all genes present in both groups, multipartite and unipartite genomes, were used as features, whereas genes unique to each group (thus eliminating the common genes) were used in the second. For each of these, a matrix was created with each row representing a sample (bacterial genome) and each column representing a gene, with presence of gene in a genome marked by ‘1’ and absence by ‘0’ in the binary matrix. The last column of the matrix is for the sample label, where multipartite is coded by ‘1’ and unipartite is coded by ‘0’. Each matrix was then used to derive three different sets, namely, ‘All Set’, ‘Intersection Set’, and ‘Random Set’, which were used for the assessment of machine learning (ML) algorithms—(i) All Set: entire gene dataset, (ii) Intersection Set: genes deemed important for discrimination by ML that appeared in all 6 rounds of the ML 6-fold cross-validation, and (iii) Random Set: randomly sampled genes (as many as in the Intersection Set) from All Set. The performance of the ML algorithms was assessed and compared by using various accuracy metrics, including F1 score, classification accuracy (for 10-fold cross-validation), area under the ROC curve, and area under the precision and recall curve.

Complete genome sequences of both the multipartite bacteria and their closest single-chromosome relatives (a total of 42 genomes) with the respective annotation files were retrieved from the NCBI RefSeq database (ftp://ftp.ncbi.nlm.nih.gov/genomes, accessed on 3 March 2020). The NCBI summary.txt was utilized to filter out incomplete assemblies and retain only the fully assembled genomes. The dataset was then processed for two experiments—(i) an all-gene-level analysis and (ii) a differentially present gene-level analysis. For the all-gene approach, a set of all the genes from both multipartite and unipartite (single chromosome) genomes was considered, and a matrix recording the presence and absence of these genes in the genomes was created. For the differentially present approach, the genes that are present in the bacteria with multipartite genomes but absent from their closest relative bacteria with single-chromosome genomes, and the genes that are absent in the bacteria with multipartite genomes but are present in their closest relative bacteria with single-chromosome genomes, were determined based on sequence alignment by using the Basic Local Alignment Search Tool (BLAST) and phylogenetic reconstruction (Almalki et al., 2022 [25]). Then, a similar second matrix on gene presence and absence was created. These matrices were used as inputs to train the ML algorithms as described below (see also Figure 1).

In addition, while we considered all the genes in these genomes, the presence or absence of the named genes in these genomes was noted, and for hypothetical protein genes, BLAST was used to identify the genes sharing high similarity in these genomes, and for each of these gene families, their presence or absence was noted in these genomes. Note that the latter was performed because the genes annotated as “hypothetical proteins” in these genomes do not have separate gene nomenclature and are commonly referred to as “hypothetical protein”. Hypothetical protein genes lacking homologs were considered as single-gene families, and their presence or absence was recorded accordingly in the matrices. These data were then organized into matrices to be input into machine learning programs; each row represents a bacterium, and each column represents a gene in the matrix. The matrix entries are binary (0 or 1). The bacterial genotypes were coded as 1 for gene presence and 0 for gene absence, and the bacterial phenotypes were coded as 0 for multipartite and 1 for unipartite in the matrix (Figure 1). The matrix data have been made available at the project’s GitHub repository at https://github.com/Janaksunuwar/Predicting-Multipartite-and-Unipartite-Bacterial-Genomes, accessed on 23 October 2023.

### 2.2. Performance Assessment of Machine Learning Methods

An assessment of the twelve machine learning algorithms’ ability to classify multipartite and unipartite traits was performed by using scikit-learn’s machine learning library for Python: http://scikit-learn.org/stable/, accessed on 22 October 2023, Version 1.2.2. The twelve machine learning algorithms assessed were Logistic Regression (logR), Gaussian Naive Bayes (gNB), Support Vector Machine (SVM), Decision Trees (DT), Random Forest (RF), K-Nearest Neighbors (KNN), Linear Discriminant Analysis (LDA), Multinominal Naive Bayes (mNB), AdaBoost Classifier (ABC), Gradient Boosting Classifier (GBC), ExtraTrees Classifier (ETC), and Bagging Classifier (BC). Accuracy metrics, namely the precision, recall, F1 score, classification accuracy for nested ten-fold cross-validation, area under the receiver operating characteristic curve (AU ROC), and area under the precision–recall curve (AUPR), were used to assess the performance of the twelve algorithms.

Their performance was assessed in three ways based on the aforementioned three gene sets: the All Set, Intersection Set, and Random Set (Figure 1). First, in the All Set performance assessment, the entire gene dataset was divided into six equal parts; each part (1/6th of the dataset) was used as a test set in turn, with the remaining parts (5/6th of the dataset) used as a training set to learn the algorithm parameters. The performance was then assessed on each test set, and finally, the overall performance was obtained by averaging the six rounds (6-fold cross-validation). Second, in the Intersection Set performance assessment, only the genes that were deemed important for classification by machine learning in each round of the aforementioned 6-fold cross-validation were considered, and an Intersection Set comprising the important genes that appeared consistently in each round of the 6-fold cross-validation was obtained. This set was then used in place of the All Set for the performance assessment in the same way that the All Set data were used. Third, in the Random Set performance assessment, as many genes as were in the Intersection Set were randomly sampled from the All Set, and then the performance was assessed by using this set. Ten such Random Sets were generated; the performance was assessed on each and then averaged to obtain the overall Random Set performance. Note that the random dataset was also divided in the same way as for the All Set data and the Intersection Set data (6-fold cross-validation), as described in the Methods section (see also Sunuwar and Azad, 2021, 2022 [23,24]). Here, the assessment was performed by using 6-fold cross-validation that entails splitting the dataset into 6 nonoverlapping equal-size sets and using each of these sets as the test set in turn with the remaining 5 sets used to train the ML model. Further, a nested 10-fold cross-validation was used for each of the six splits, and then the performance (accuracy) was obtained as the average over these.

### 2.3. Generating Gene Sets of Differentially Present (or Absent) Genes Using the Genes Deemed Important in Discrimination by Machine Learning

We segregated the genes that were deemed important for discrimination into 3 sets: Set 1 contains the genes that are present in bacteria with multipartite genomes (a large majority or most or all) but are absent in their closest relative bacteria with single-chromosome genomes; Set 2 contains the genes that are absent in bacteria with multipartite genomes but are present in their closest relative bacteria with single-chromosome genomes (a large majority or most or all); and Set 3 contains the genes that do not belong to Set 1 and Set 2. We used BLAST to generate these gene sets; the homology or similarity inference was based on an E-value threshold of 10^−5^, and additionally, a >70% query coverage and >30% identity were required (Altschul et al., 1990 [26]). This was performed for both approaches; that is, when all the genes in all 42 genomes were considered as well as when only the differentially present genes as established by the phylogenetic approach (Almalki et al., 2022 [25]) were considered.

### 2.4. Principal Component Analysis (PCA)

A dimensionality reduction by Principal Component Analysis (PCA) was performed by using scikit-learn to visualize the multipartite and unipartite genomes on the two-dimensional projection of the gene space. A two-component PCA for the features (genes) that appeared consistently in all six rounds of cross-validation, i.e., the Intersection Set genes, was performed. Data standardization was performed by using scikit-learn’s standard scaler, and a fit transform was applied.

### 2.5. Determination of Gene Function Using eggNOG

We prepared fasta files for all the gene sets that were obtained from the all-genes approach as well as from the differentially present genes approach, and we then used the eggNOG program (Huerta-Cepas et al., 2017 [27]) to obtain gene function annotation in the gene sets. We used both orthology restrictions, that is, transfer annotation from any ortholog and transfer annotation from one-to-one orthology only, as well as bacteria as the taxonomic scope.

## 3. Results and Discussion

### 3.1. Assessment of Performance of Different Machine Learning Algorithms

**Figure 2:**
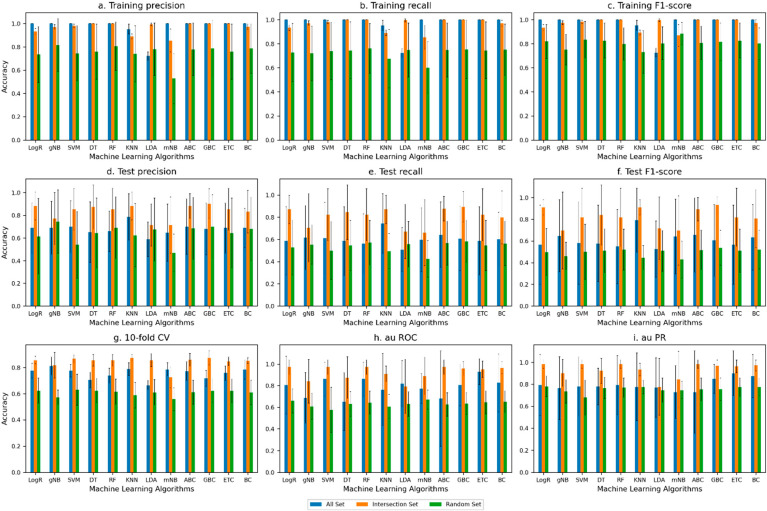
Assessment of the performance of the machine learning algorithms in classifying multipartite and unipartite genomes based on gene-level analysis under 6-fold cross-validation setting; here, to begin with, all genes in the multipartite and unipartite genomes were considered. The performance metrics used were (**a**) training precision, (**b**) training recall, (**c**) training F1 score, (**d**) test precision, (**e**) test recall, (**f**) test F1 score, (**g**) 10f CV (ten-fold cross-validation), (**h**) AU ROC (area under ROC curve), and (**i**) AU PR (area under precision–recall curve). ‘All Set’ denotes all genes for training (as in the cross-validation partitioning), ‘Intersection Set’ refers to set of genes that consistently ranked high across all 6 rounds of cross-validation, and ‘Random Set’ refers to randomly sampled genes.

**Figure 3:**
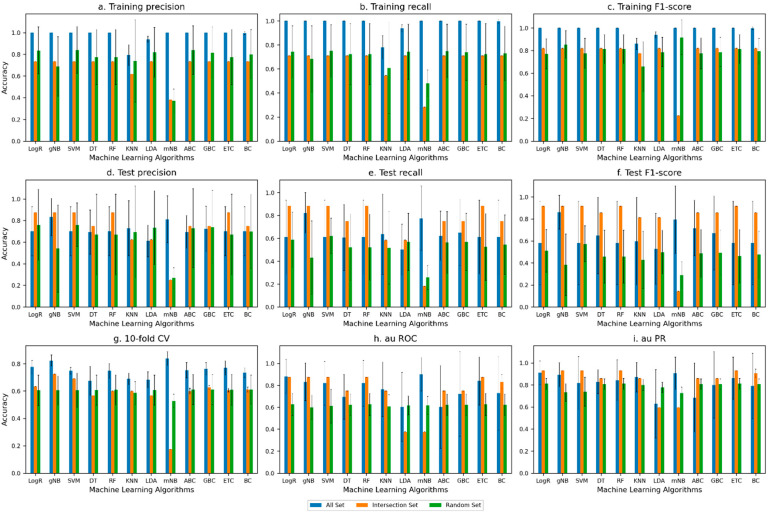
Assessment of the performance of the machine learning algorithms in classifying multipartite and unipartite genomes based on differentially present gene-level analysis under 6-fold cross-validation setting. The performance metrics used were (**a**) training precision, (**b**) training recall, (**c**) training F1 score, (**d**) test precision, (**e**) test recall, (**f**) test F1 score, (**g**) 10f CV (ten-fold cross-validation), (**h**) AU ROC (area under ROC curve), and (**i**) AU PR (area under precision–recall curve). ‘All Set’ denotes all genes for training (as in the cross-validation partitioning), ‘Intersection Set’ refers to set of genes that consistently ranked high across all 6 rounds of cross-validation, and ‘Random Set’ refers to randomly sampled genes.

The performance of the machine learning methods on the All Set and Intersection Set showed that the methods achieved better overall accuracy (measured by using the F1 score) with the Intersection Set (the test F1 score in Figure 2 and Figure 3). Figure 2 shows the performance for the all-genes approach (when all the genes in all the 42 genomes were considered), and Figure 3 shows the performance for the differentially present genes approach (when only the genes that are differentially present/absent in multipartite versus unipartite genomes were considered, which were obtained by using a BLAST phylogenetic analysis). The performance with the Intersection Set was overall superior for both approaches (the performance on the test set was quantified by using recall, precision, the F1 score, AU ROC, and AU PR in Figure 2 and Figure 3). Performances on the All Set and Intersection Set were substantially better than the performance on the Random Set, as expected.

Close to a 92% test F1 score on the Intersection Set was achieved with both the all-genes approach (Figure 2) and the differentially present genes approach (Figure 3), with Logistic Regression (logR), Support Vector Machine (SVM), Random Forest (RF), K-Nearest Neighbors (KNN), Decision Trees (DT), ExtraTrees Classifier (ETC), Gradient Boosting Classifier (GBC), and Bagging Classifier (BC) attaining this level of performance in the former (Figure 2) and Gaussian Naive Bayes (gNB) in the latter (Figure 2). The lowest recorded F1 score was for the Random Set, as expected.

Note that the high-ranked genes obtained from the All Set approach were used to construct the Intersection Set. These genes consistently appeared among the top-ranked genes in all six rounds of the six-fold cross-validation. The high accuracy with the Intersection Set suggests that these genes are indeed the most informative in discriminating multipartite and unipartite genomes and could thus be the drivers of the evolution of such traits. These genes helped the most in predicting the bacteria with a multipartite genome and the bacteria with a unipartite genome, just based on the gene sets.

In addition to the aforementioned accuracy metrics used in the assessment (see also Figure 2 and Figure 3), we also used the Matthew Correlation Coefficient (MCC) to evaluate the performance of the ML models on the Intersection Set. The MCC value of +1 indicates that the classifier made no mistakes, a 0 value indicates average random prediction, and a −1 value indicates disagreement between the predictions and observations. 

In the all-gene-level analysis, KNN and GBC have the highest MCC (0.787 and 0.741, respectively) amongst all. Both GBC and KNN also produced high F1-score values (0.93 and 0.91, respectively), and additionally, LogR and RF also yielded an F1 score of 0.91. In the differentially present gene-level analysis, the gNB-, LogR-, and SVM-generated MCC values of 0.508, 0.442, and 0.475, respectively, were higher than the other ML models, indicating a moderate-to-strong correlation (agreement). For comparison, the F1-score values of these classifiers in this analysis were the same at 0.917; additionally, RF and ETC yielded this level of performance (Supplementary Table S1).

In general, in the all-gene approach, tree-based classifiers, namely GBC, RF, and ETC, have relatively high MCC values, with GBC standing out with both the MCC and F1 score higher than the other ML classifiers. In the differentially present gene approach, the MCC values for the tree-based classifiers were relatively lower, unlike the F1-score values, especially for RF and ETC. Note that previous studies have reported a decline in the MCC for imbalanced datasets (Zhu, 2020 [28]). Taken together, the tree-based classifiers performed comparably well in discriminating multipartite and unipartite genomes.

### 3.2. Principal Component Analysis 

**Figure 4:**
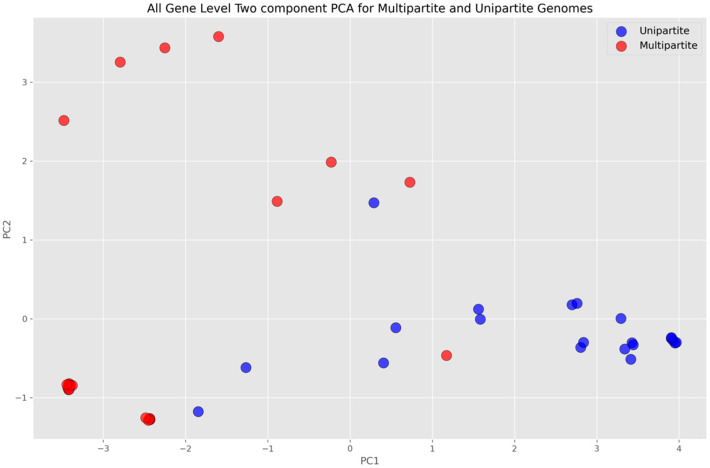
All-gene-level PCA plot, where blue dots represent unipartite genomes and red dots represent multipartite genomes. The horizontal axis represents the first component and vertical axis represents the orthogonal of first and second components. The genes that appeared consistently in all six rounds of cross-validation were standard-scaled and fit-transformed to perform the analysis.

**Figure 5:**
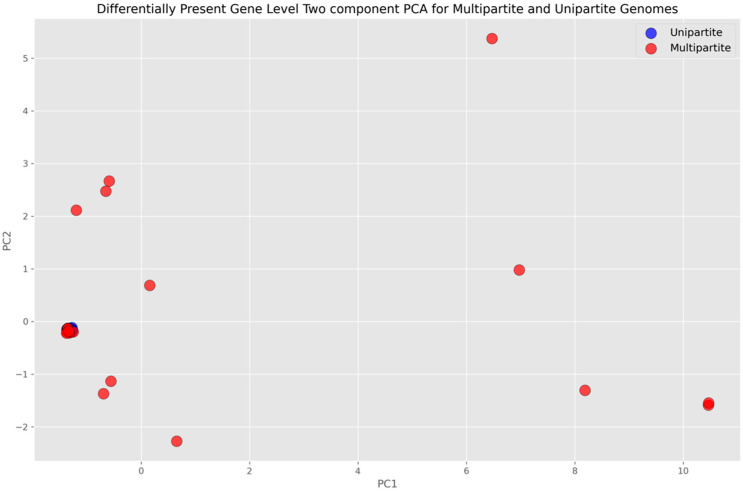
Differentially present gene-level PCA plot, where blue dots represent unipartite genomes and red dots represent multipartite genomes. The horizontal axis represents the first component and vertical axis represents the orthogonal of first and second components. The genes that appeared consistently in all six rounds of cross-validation were standard-scaled and fit-transformed to perform the analysis.

PCA was performed for both all-gene and differentially present gene-level analyses. The multipartite and unipartite genomes were mapped on the PCA space with dimensions corresponding to the number of genes that were deemed important (discriminatory) by the ML algorithms and consistently selected in each round of the cross-validation process. The projection of this space onto a two-dimensional plane by using PCA (characterized by first and second principal components) is shown in Figure 4 for the all-gene approach and in Figure 5 for the differentially present gene approach. A clear distinction between the multipartite and unipartite genomes is discernible in the former, with the multipartite genomes localized mostly towards the upper partition and the unipartite genomes mostly localized towards the lower partition of the plane by the diagonal (Figure 4), whereas in the latter, the unipartite appeared coalesced at a single locus in contrast to the multipartite, with the distinction between the two not clearly demarcated in the two-dimensional principal component representation (Figure 5) as it is in the former (Figure 4).

### 3.3. Gene Sets Derived from Genes That Consistently Ranked High across All Rounds of the Cross-Validation Gene Sets

As described in the Methods section, following obtaining the genes that were deemed important for discrimination in classifying multipartite and unipartite genomes by the ML algorithms (top 15 such genes are provided in Table 1 from all-gene-level analysis and in Table 2 from differentially-present-gene-level analysis), three gene sets (Set 1, Set 2, and Set 3) were generated for the all-genes approach as well as for the differentially-present-genes approach. We found from our analysis that of the 46 genes obtained by using the all-genes approach, 5, 22, and 19 were categorized under Set 1, Set 2, and Set 3, respectively (Table 3). We similarly apportioned 35 genes obtained by using the differentially present genes approach into three sets, with 11 assigned to Set 1, 12 to Set 2, and 12 to Set 3 (Table 4). 

### 3.4. Functional Annotation of Genes Obtained from All-Genes Approach and from Differentially Present Genes Approach Using eggNOG

We used the eggNOG program to assess the function of the genes in the aforementioned three gene sets obtained from both the all-genes and differentially present genes approaches (Table 3 and Table 4). We focused on Set 1 and Set 2, with the former containing genes that are present in most of the multipartite genomes but not in their unipartite close relatives and the latter containing genes that are present in most of the unipartite close relatives of multipartite genomes but not in the multipartite genomes. For the all-gene approach, in Set 1, four genes (80%) were annotated under metabolism, specifically coenzyme transport and metabolism (H) for 3-octaprenyl-4hydroxybenzoate carboxy-lyase and amino acid transport and metabolism (E) for aminopeptidase p, dihydrodipicolinate synthase, and prephenate dehydratase genes. On the other hand, one gene (20%), 30s ribosomal protein s17, was classified as translation, ribosomal structure, and biogenesis (J), which is under information storage and processing. For Set 2, one gene (~5%) was annotated with an unknown function. Seven genes (~32%) were categorized under metabolism, namely acyl-coa dehydrogenase domain-containing protein, cytochrome p450 family protein, fad dependent oxidoreductase, inorganic polyphosphate/atp-nad kinase, nitroreductase family protein, thiamine-phosphate kinase, and lysophospholipase l2. Five genes (~23%) were annotated under information storage and processing, namely gtp-dependent nucleic acid-binding protein and s1 rna binding domain protein (categorized as translation, ribosomal structure, and biogenesis), cupin domain protein, transposase is3/is911 family protein, and transposase is4 family protein (the latter three were categorized as replication, recombination, and repair (L)). Seven genes (~32%) were annotated under cellular processes and signaling, namely 10 kda chaperonin; cyclic nucleotide-binding domain protein; mate efflux family protein; pii uridylyl-transferase; ribosome biogenesis gtp-binding protein; universal stress family protein; and the efflux transporter, rnd family, and mfp subunit. One gene (~5%), a transporter that belongs to the cpa2 family, was annotated with two functions—signal transduction mechanisms and inorganic ion transport and metabolism (PT). Here, it is obvious that most of the genes in Set 1 are categorized under metabolism, and that could be explained by the lifestyle of the bacteria with multipartite genomes; for example, the fact that they live in extreme conditions or are pathogenic, which may necessitate the presence of disproportionally more metabolic genes (Table 3).

On the other hand, we found the following proteins in the Set 1 genes by using the differentially present genes approach (Table 4): cell division protein ftsz, diguanylate cyclase/phosphodiesterase, and ompa/motb domain protein. These were categorized under cellular processes and signaling. More specifically, these genes have cell-cycle control; cell division; a chromosome-partitioning function (D); signal transduction mechanisms (T); and cell wall, membrane, and envelope biogenesis (M). Also, mannosylglycerate synthase domain protein-encoding gene in this set was categorized under metabolism, and more specifically, nucleotide transport and metabolism (F). Another gene in this set encodes a putative metal-dependent protease that has a replication, recombination, and repair function (L), and more broadly, it is categorized under information storage and processing. In addition to these, three genes were categorized under “unknown function” by the eggNOG program: the cobalamin biosynthesis protein CbiG, extensin family protein, and a protein of unknown function duf1127. Three genes, namely those encoding the gcra cell-cycle regulator, invasion-associated locus b family protein, and HPS_22 (hypothetical protein), were not scanned by the eggNOG program. 

For Set 2 from the differentially present genes approach, we found the following functional representation (Table 4): genes encoding d-isomer-specific 2-hydroxyacid dehydrogenase and nad-binding protein (CH) were functionally classified as energy production and conversion and coenzyme transport and metabolism, both under the category of metabolism. Additionally, gene-encoding glycine dehydrogenase subunit 2 is associated with amino acid transport and metabolism (E), which is also under metabolism. On the other hand, the genes encoding preprotein translocase, the secg subunit, and the outer membrane chaperone skp family protein were categorized under cellular processes and signaling, and the specific functions are intracellular trafficking; secretion and vascular transport (U); and cell wall, membrane, and envelope biogenesis (M), respectively. Three genes were categorized under unknown function, namely those encoding the protein of unknown function duf389, tpr repeat domain protein, and twin-arginine translocation pathway signal domain protein. Four genes encoding hypothetical proteins were not scanned by the eggNOG program (Table 4).

One notable observation was that the presence or absence of metabolic genes differentiates multipartite genomes and their close single-chromosome-genome relatives. Specialized metabolic genes were found in multipartite genomes but not in their close unipartite relatives, and similarly, certain metabolic genes were found to be absent in multipartite genomes, but these were found in the genomes of their close unipartite relatives. Clearly, metabolic genes appear to be an important factor in the evolution of multipartite genomes. Our analysis also revealed that certain genes that are involved in cell-cycle control, cell division, and chromosome-partitioning functions are harbored by multipartite genomes but not by their close unipartite relatives (Table 3 and Table 4). These findings suggest that these genes might have a role in the emergence of multipartite genomes, likely from their unipartite genome ancestors. Further studies are needed to understand these genes and how their networks facilitate the emergence, selection, and adaptation of bacteria with multipartite genomes. 

## 4. Conclusions

Machine learning has become a powerful tool, particularly in dealing with big data, such as large-scale genomic data. Here, we used it as a means to identify genetic factors that discriminate, in general, between bacteria with multipartite genomes and bacteria with unipartite genomes. Our results demonstrate the ability of several machine learning methods to predict multichromosome traits in bacteria based on their gene content. The overall accuracy was ~90%, and this motivated us to identify the genes that aided in the discrimination between multipartite and unipartite bacteria. We used feature selection by machine learning methods to identify the features (genes), ranked by their importance, that aided in the prediction of unipartite and multipartite traits. This yielded 46 genes via the all-gene approach and 35 genes via the differentially present genes approach. We performed a sequence alignment using BLAST for these genes against all the genomes included in this study. Based on the BLAST results, we assigned the genes deemed important in discrimination to three lists. Set 1 included the genes that were in a large majority (or most or all) of the bacteria with multipartite genomes but not in the unipartite bacteria, and Set 2 included the genes that were in a large majority (or most or all) of the closest relatives of the multipartite bacteria that had a single-chromosome genome but not in the multipartite bacteria. We assessed the functions of the genes in these sets and found that both contain numerous genes with functions associated with metabolism. Note that we reported similar results in a phylogeny-based study (Almalki et al., 2022 [25]) where we investigated the functions of the genes gained in bacteria with multipartite genomes. Our analysis demonstrates that machine learning is an effective tool for inferring the genes underlying the differentiation of multipartite and unipartite bacterial genomes and could aid in our understanding of the emergence of differential traits in bacteria.

## Figures and Tables

**Table 1:** List of top 15 genes deemed important or discriminative by machine learning algorithms in classifying multipartite and unipartite genomes based on all-gene-level analysis.

	Most Important Features for Gene-Level Analysis
1	transposase, IS4 family
2	MATE efflux family protein
3	dihydrodipicolinate synthase
4	metallo-beta-lactamase family protein
5	tRNA pseudouridine synthase A
6	transcriptional regulator, AraC family
7	dihydrodipicolinate reductase
8	glyoxalase family protein
9	efflux transporter, RND family, MFP subunit
10	OsmC-like protein
11	transcriptional regulator, MarR family
12	riboflavin biosynthesis protein RibF
13	tRNA pseudouridine synthase B
14	FeS assembly ATPase SufC
15	cyclic nucleotide-binding domain protein

**Table 2:** List of top 15 genes deemed important or discriminative by machine learning algorithms in classifying multipartite and unipartite genomes based on differentially-present-gene-level analysis.

	Most Important Features for Differentially Present Gene-Level Analysis
1	transcriptional regulator
2	chemotaxis protein
3	sugar ABC transporter permease
4	flagellar M-ring protein FliF
5	acetolactate synthase
6	PAS domain-containing protein
7	sugar ABC transporter substrate-binding protein
8	Porin
9	cell envelope biogenesis protein TolA
10	hybrid sensor histidine kinase/response regulator
11	GntR family transcriptional regulator
12	chemotaxis protein CheD
13	2-C-methyl-D-erythritol 4-phosphate cytidylyltransferase
14	short-chain dehydrogenase
15	2-C-methyl-D-erythritol 2,4-cyclodiphosphate synthase

**Table 3:** Gene annotations for Set 1, Set 2, and Set 3 obtained by using the all-genes approach. Set 1 contains the genes that are present in bacteria with multipartite genomes (a large majority or most or all) but are absent in their closest relatives with single-chromosome genomes; Set 2 contains the genes that are absent in bacteria with multipartite genomes but are present in their closest relatives with single-chromosome genomes (a large majority or most or all); and Set 3 contains the genes that do not belong to Set 1 and Set 2.

Set 1	Set 2	Set 3
3-octaprenyl-4hydroxybenzoate carboxy-lyase (H)	10 kda chaperonin (O)	farnesyltranstransferase (H)
30s ribosomal protein s17 (J)	acyl-coa dehydrogenase domain-containing protein (I)	maf-like protein (D)
aminopeptidase p (E)	cyclic nucleotide-binding domain protein (T)	nad-dependent deacetylase (K)
dihydrodipicolinate synthase (E)	cytochrome p450 family protein (Q)	outer membrane efflux protein (MU)
prephenate dehydratase (E)	fad dependent oxidoreductase (Q)	prevent-host-death family protein (J)
	gtp-dependent nucleic acidbinding protein engd (J)	ribosomal large subunit pseudouridine synthase d (-)
	inorganic polyphosphate/atpnad kinase (G)	thymidine kinase (F)
	mate efflux family protein (V)	transcriptional regulator, padr family (K)
	nitroreductase family protein (C)	anthranilate synthase component I (E)
	pii uridylyl-transferase (O)	cation diffusion facilitator family transporter (P)
	protein of unknown function duf403 (S)	fad-dependent pyridine nucleotide-disulfide oxidoreductase (S)
	ribosome biogenesis gtpbinding protein ysxc (D)	inositol monophosphatase family protein (G)
	s1 rna binding domain protein (J)	preprotein translocase, sece subunit (U)
	thiamine-phosphate kinase (H)	preprotein translocase, yajc subunit (U)
	transporter, cpa2 family (PT)	transcriptional regulator, arac family (K)
	universal stress family protein (T)	transcriptional regulator, merr family (K)
	HPS_21876 (-)	transcriptional regulator, tetr family (K)
	cupin domain protein (L)	trna pseudouridine synthase a (J)
	efflux transporter, rnd family, mfp subunit (M)	protein of unknown function duf482 (-)
	lysophospholipase l2 (I)	
	transposase is3/is911 family protein (L)	
	transposase, is4 family (L)	

**Table 4:** Gene annotations for Set 1, Set 2, and Set 3 obtained by using the differentially present genes approach. Set 1 contains the genes that are present in bacteria with multipartite genomes (a large majority or most or all) but are absent in their closest relatives with single-chromosome genomes; Set 2 contains the genes that are absent in bacteria with multipartite genomes but are present in their closest relatives with single-chromosome genomes (a large majority or most or all); and Set 3 contains the genes that do not belong to Set 1 and Set 2.

Set 1	Set 2	Set 3
cell division protein ftsz (D)	d-isomer-specific 2-hydroxyacid dehydrogenase, nad-binding protein (CH)	chloramphenicol acetyltransferase (V)
cobalamin biosynthesis protein cbig (S)	glycine dehydrogenase subunit 2 (E)	dna protecting protein dpra (LU)
diguanylate cyclase/phosphodiesterase (T)	hypothetical protein cp97_01065 (-)	flagellar protein flgj (MNO)
extensin family protein (S)	hypothetical protein turpa_2028 (-)	gtp-binding proten hflx (S)
gcra cell-cycle regulator (-)	outer membrane chaperone skp family protein (M)	lipoprotein, putative (S)
Invasion-associated locus b family protein (-)	preprotein translocase, secg subunit (U)	stage ii sporulation protein e (T)
mgs domain protein (F)	protein of unknown function duf389 (S)	transcriptional regulator, crp/fnr family (K)
ompa/motb domain protein (M)	single-stranded dna-binding protein-1 (L)	transcriptional regulator, merr family (K)
protein of unknown function duf1127 (S)	tpr repeat domain protein (S)	transcriptional regulator, tetr family (K)
putative metal-dependent protease (L)	twin-arginine translocation pathway signal domain protein (S)	hypothetical protein cp97_01545 (-)
HPS_22 (-)	HPS_105 (-)	hypothetical protein cp97_01880 (-)
	HPS_107 (-)	hypothetical protein cp97_06070 (-)

## Data Availability

Custom programs, Jupyter notebooks, and the associated datasets have been made available at https://github.com/Janaksunuwar/Predicting-Multipartite-and-Unipartite-Bacterial-Genomes, accessed on 23 October 2023.

## Supplementary Materials

The following supporting information can be downloaded at: https://www.mdpi.com/article/10.3390/microorganisms11112756/s1, Table S1: Matthews Correlation Coefficient for all Gene Level and Differentially Present Gene Level for the final Machine Learning Algorithms trained on Intersection Set in predicting Multipartite and Unipartite Genomes.
