# Supplementary material for: Using Machine Learning to Predict Genes Underlying Differentiation of Multipartite and Unipartite Traits in Bacteria
Source: Microorganisms. 2023 Nov 13;11(11):2756. doi: 10.3390/microorganisms11112756 (PMC10672838; doi:10.3390/microorganisms11112756)
Supplement: Supplementary file 1 [file microorganisms-11-02756-s001.zip › microorganisms-2512335-supplementary.pdf]

Supplementary Table S1. Matthews Correlation Coefficient for all Gene Level and Differentially Present Gene Level for the final Machine Learning Algorithms trained on Intersection Set in predicting Multipartite and Unipartite Genomes.

		**All Gene Level**		**Differentially Present Gene Level**	
SN.	Classifier	MCC	F1 Score	MCC	F1 Score
**1**	LogR	0.619	0.91	0.442	0.917
**2**	gNB	0.583	0.696	0.508	0.917
**3**	SVM	0.619	0.818	0.475	0.917
**4**	DT	0.633	0.839	0.331	0.857
**5**	RF	0.708	0.818	0.331	0.917
**6**	KNN	0.787	0.91	0.272	0.812
**7**	LDA	0.477	0.716	0.240	0.812
**8**	mNB	0.359	0.697	-0.200	0.143
**9**	ABC	0.532	0.893	0.377	0.857
**10**	GBC	0.741	0.93	0.409	0.857
**11**	ETC	0.653	0.818	0.409	0.917
**12**	BC	0.500	0.806	0.298	0.857
